# Chemi-Ionization Reactions and Basic Stereodynamical Effects in Collisions of Atom-Molecule Reagents

**DOI:** 10.1021/acs.jpca.1c00688

**Published:** 2021-04-15

**Authors:** Stefano Falcinelli, Franco Vecchiocattivi, James M. Farrar, Fernando Pirani

**Affiliations:** † Department of Civil and Environmental Engineering, University of Perugia, Via G. Duranti 93, 06125 Perugia, Italy; ‡ Department of Chemistry, University of Rochester, 14627 Rochester, New York, United States; § Department of Chemistry, Biology and Biotechnologies, University of Perugia, Via Elce di Sotto 8, 06123 Perugia, Italy

## Abstract

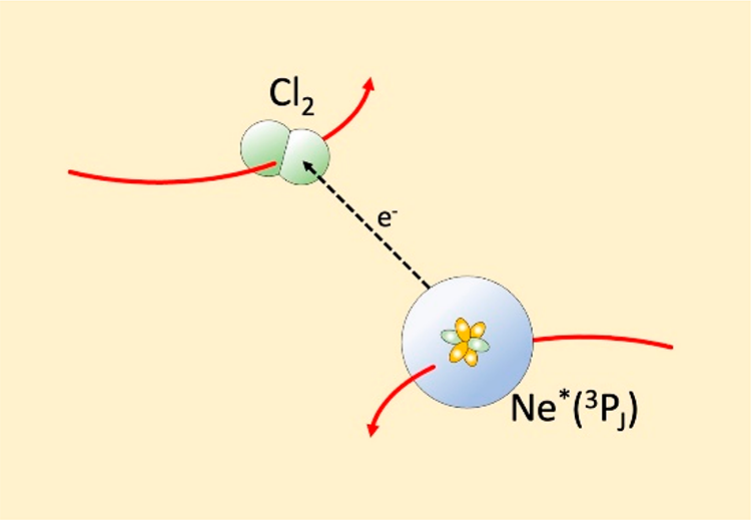

A new theoretical method, developed by our laboratory to describe the microscopic dynamics of gas-phase elementary chemi-ionization reactions, has been applied recently to study prototype atom–atom processes involving reactions between electronically excited metastable Ne*(^3^P_2,0_) and heavier noble gas atoms. Important aspects of electronic rearrangement selectivity have been emphasized that suggested the existence of two fundamental microscopic reaction mechanisms. The distinct mechanisms, which are controlled by intermolecular forces of chemical and noncovalent nature respectively, emerge under different conditions, and their balance depends on the collision energy regime investigated. The present paper provides the first step for the extension of the method to cases involving molecules of increasing complexity, whose chemi-ionization reactions are of relevance in several fields of basic and applied researches. The focus is here on the reactions of Ne* with simple inorganic molecules as Cl_2_ and NH_3_, and the application of the method discloses relevant features of the reaction microscopic evolution. In particular, this study shows that the balance of two fundamental reaction mechanisms depends not only on the collision energy and on the relative orientation of reagents but also on the orbital angular momentum of each collision complex. The additional insights so emphasized are of general relevance to assess in detail the stereodynamics of many other elementary processes.

## Introduction

The weakly bound adducts formed by colliding reagents play an important role in the kinetics of elementary processes, serving as precursor states opening the passage to the transition state (TS) of several chemical–physical phenomena occurring in gaseous and condensed phases and at the gas–solid, gas–liquid interphases.^1−4^ The valence electrons quantum confinement of reagents in such adducts and the selectivity of electronic rearrangements, promoting the stereodynamical evolution of the systems toward the final products, is still today a topic of general interest.

Recently, we developed and applied a new/original method^5−7^ to the detailed investigation of chemi-ionization reactions (CHEMI), promoted by collisions of atoms electronically excited in metastable states with other atoms, that are prototypical of barrierless processes leading to the formation of ionic products plus electrons.^8−10^ The study of them remains of great interest for fundamental and applied research, since it allows a definition of the role of the barrierless reactions in cold chemistry^11−13^ and an opportunity to model energy-transfer phenomena occurring in flames, plasmas, and electric discharges.^14,15^

If CHEMI involving Ng atoms are important, especially from the point of view of basic research, those involving molecules are of a more general interest, especially in highlighting the role of electronic transfer, that is, the redox nature of this type of process.^6,7^ Indeed, they control the balance of phenomena occurring in interstellar environments, in combustion and flames, where CHEMI are considered as the primary initial step,^14,15^ in molecular plasmas and nuclear fusion. They also govern interstellar chemistry and planetary ionospheres^16−18^ affecting the transmission of radio and satellite signals.^18^ These reactions are also implicated in soft-ionization mass spectrometry techniques,^19,20^ since the controlled internal degrees excitation of the molecular ionic products limits the number of fragmentation channels.

In the present study we attempt to take the first step toward the extension/generalization of our approach to atom-molecule CHEMI, where the intermolecular interactions driving the dynamics are usually stronger and more anisotropic with respect to atom–atom CHEMI and often include further components. The combination of these interaction features can strongly vary the relative role of two basic (*direct* and *indirect*) mechanisms, initially demonstrated for atom–atom CHEMI reactions.^5−7^

In the next sections, after a comparison of the experimental total ionization cross sections, with their different magnitudes and collision energy dependence, the focus is on some prototypical CHEMI of molecules. The special case of Cl_2_ emphasizes how the typical harpooning effect can affect the reaction precursor state. The detailed study of NH_3_ reaction stereodynamics shows how the two basic mechanisms, first revealed for atom–atom CHEMI, are modulated by molecular orientation and by the orbital angular momentum of the collision complex, controlling the centrifugal component of the total interaction potential.

## Methods

Our investigation, based on a new semiclassical treatment fully described in recent papers,^5−7^ focused on Ne*(^3^P_2,0_)-Ng (Ng = Ar, Kr, Xe) systems, and the analysis provided an internally consistent rationalization of available experimental findings, such as Penning ionization electron spectra (PIES), total and partial ionization cross sections, and their branching ratios (BRs). The detailed characterization of the atom–atom reaction dynamics revealed new insights into the role of rearrangement and the angular momentum coupling of valence electrons in chemical kinetics that must be considered of general interest for many other reactions. In particular, the application of the method^5−7^ suggests that

(1) The optical potential model, formally introduced to describe nuclear reactions dynamics and applied also to CHEMI,^9,10^ is defined as a combination of a real and an imaginary part. We have demonstrated^5−7^ that the two parts—that control, respectively, the collision dynamics and the “opacity” or probability of CHEMI—must be interdependent, since they arise from the same interaction components.
(2) The different balance of such components originates two competitive microscopic reaction mechanisms. They have been identified, respectively, as a *direct mechanism*, dominant at short separation distances of reagents being driven by chemical forces, and an *indirect mechanism*, prevalent at large separation distances and originating from noncovalent forces, such as dispersion, induction-polarization contributions, and those promoting spin–orbit and centrifugal-Coriolis effects.^5−7^ In particular, the *direct mechanism* is triggered by effective charge (electron) transfer (CT) effects between reagents favored by the overlap of valence orbitals. The *indirect mechanism* describes ionization that occurs also by a concerted emission-absorption of a “virtual” photon exchanged by reagents within the confines of the weakly bound collision complex. Therefore, while the *direct mechanism* controls the evolution of prototype elementary *oxidation reaction*s, the *indirect mechanism* triggers typical *radiative* (*photo)-ionization processes*.^7^
(3) The reactivity depends on the collision energy (*E*_coll_), separation distance *R*, and relative alignment of valence orbitals, important factors that affect the structure and stability of the adducts formed by collision of reagents and then of the reaction TS.
(4) Twelve reaction channels, ascribed to specific passages from a quantum state of reagents to that of products, have been characterized, where each one is affected by a different relative role of the two basic mechanisms mentioned above.^7^

## Results and Discussion

### General Trends

**Figure 1:**
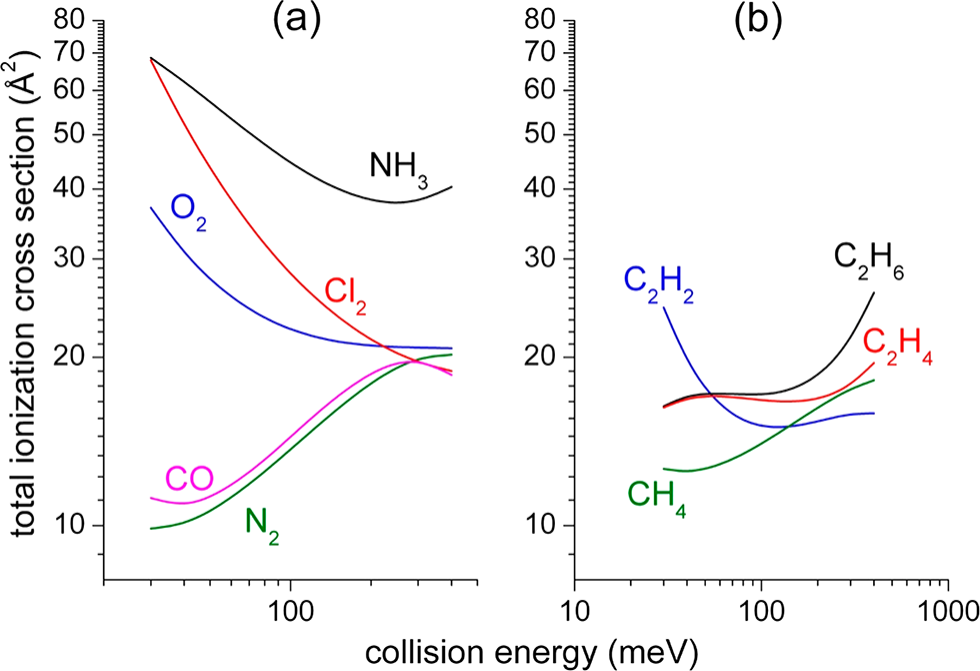
Total ionization cross sections in some Ne*-molecule systems, as a function of collision energy. The curves are interpolating 3rd degree polynomials of experimental data.^22,25^ (a) The case of some inorganic molecules. (b) The case of the simplest saturated and unsaturated hydrocarbons.

According to the pioneering work of Beijerinck and co-workers,^21^ molecular systems giving CHEMI, all experimentally investigated in detail in the gas phase under single collision conditions with the molecular beam technique, can be distinguished in two groups: CHEMI systems showing a pronounced increase in the total ionization cross section as *E*_coll_ increases and CHEMI systems showing, contrariwise, cross sections with a decreasing trend. The energy dependence of the total ionization cross section has been measured in our laboratory in an internally consistent way for several systems involving Ne*;^22−25^ therefore, a direct and quantitative comparison of obtained results is straightforward. Some prototype examples are reported in Figure 1a,b.

**Figure 2:**
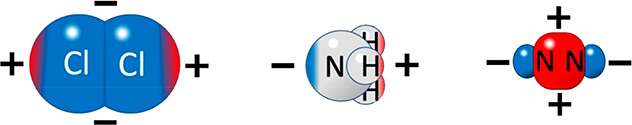
Fundamental features of Cl_2_, NH_3_, and N_2_ molecules associated with different electronic charge distribution around their molecular axis. The chlorine molecule exhibits two σ-holes collinear with the bond axis. This justifies the large and positive quadrupole moment of Cl_2_. The ammonia molecule exhibits a large dipole moment. The nitrogen molecule exhibits a large and negative quadrupole moment. The positive charge density increase is approximately indicated by the increased extent of the red color; the corresponding change in negative charge density is likewise indicated in blue.

The differing behaviors, exhibited by the various partners of the Ne* reagent, must selectively depend on their fundamental chemical physical properties, as depicted in Figure 2 for three cases of inorganic molecules. The present focus is on Ne*-Cl_2_, where the formation by harpooning of an effective ion pair is expected to increase the binding energy in the collision complex, at least 2 orders of magnitude with respect to that in Ne*-N_2_, favoring a closer approach of reagents. However, in addition to specific features of Ne*-Cl_2_, here we also analyze in detail the Ne*-NH_3_ system for which the intermolecular interaction is strongly anisotropic, exhibits an intermediate strength between Ne*-Cl_2_ and Ne*-N_2_, and has been recently provided in analytical form (see below).

### The Ne*-Cl_2_ Case

To cast light on the critical role of the interaction components that are expected to selectively modulate the relative weight of the two basic microscopic mechanisms indicated above as a function of collision energy, we make a preliminary attempt to rationalize the phenomenology observed for the Ne*-Cl_2_ system shown in Figure 1a. In particular, in the thermal range of *E*_coll_, Ne*-Cl_2_ is one of the systems showing the highest cross-section value. For the Cl_2_ reagent this behavior must relate to the electronic features of its structure. The potential curves for the ground and excited states of the chlorine molecule as well as of its positive and negative ions have been calculated by Peyerimhoff and Buenker using the MRD-CI method,^26^ to which the interested reader can refer. Specifically, this molecule exhibits a high permanent electric quadrupole moment (+3.8 au),^27^ a high electron affinity (2.44 eV),^28^ and is characterized by a σ-hole,^29^ with a positive electrostatic potential confined along the outer parts of the Cl–Cl bond (see Figure 2). An extended discussion on the σ-hole topic, in terms of electron density plots of Cl_2_ molecule in its ground electronic state, is presented in ref (29) (see also references therein).

**Figure 3:**
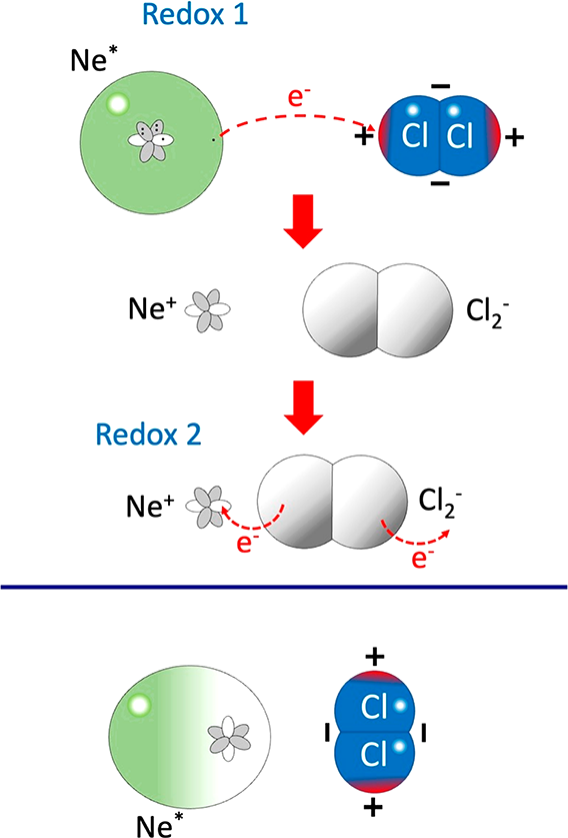
A schematic diagram representing the microscopic dynamics for Ne*-Cl_2_ CHEMI reaction. Redox 1 (upper panel) At a large distance (∼6 Å) and with collinear Cl_2_ the Rydberg electron of Ne* can go to fill the σ-hole of Cl_2_ with subsequent Ne^+^-Cl_2_^–^ ion pair formation. The extra electron in Cl_2_^–^ is located in the antibonding 3σ_u_* orbital. Redox 2 (middle panel) At shorter distances the ionization can take place involving a pair of electrons from the 3σ molecular orbitals. In this case, the Cl_2_^+^ ion is then formed in the dissociative B^2^Σ_g_^+^. (lower panel) In case of a perpendicular approach, the Ne* atom is polarized, and the ionization can take place essentially through a radiative (physical-photoionization) mechanism at a large distance, or by an exchange (chemical-redox) mechanism at a short distance. The positive charge density increase is approximately indicated by the increased extent of the red color; the corresponding change in negative charge density is likewise indicated in blue.

These unique features of Cl_2_ are indeed responsible of the formation of the intermolecular halogen bond even with lighter Ng atoms in their ground electronic state.^29^ Therefore, during the approach to Cl_2_, the “floppy” outer electronic cloud of Ne* tends to be polarized by the long-range intermolecular interaction field. This electron transfer is primarily triggered in the collinear approach by the σ-hole presence. The newly formed Cl_2_^–^ anion tends further to align with its axis along the interatomic Ne···Cl···Cl separation **R**, and the Coulomb attraction in the nascent ion-pair Ne^+^-Cl_2_^–^ favors the trapping of reagents, shown schematically in Figure 3. Indeed, in Cl_2_^–^ the added electron, populating the outer 3σ_u_* antibonding orbital, confined in the external part of the Cl–Cl bond, completely fills the σ-hole and strongly reduces the molecular bond strength making Cl_2_^–^ a highly unstable species, especially in the presence of Ne^+^.

The increased attraction arising from ion pair formation and the instability of Cl_2_^–^ stimulates the formation of a highly excited NeCl* adduct that autoionizes leading to Ne + Cl^+^ + e^–^ products. At a short *R*, an additional electronic rearrangement process become possible, illustrated in the middle panel in Figure 3, triggered by the overlap between the half-filled orbital of Ne^+^ and the populated 3σ molecular orbitals of Cl_2_. Specifically, this overlap promotes a single electron transfer from the outer 3σ_u_* of Cl_2_^–^ to the half-filled orbital of the Ne^+^ core, which is accompanied by an energy release sufficient to eject one of two electrons populating the 3σ_g_ bonding molecular orbital of Cl_2_. As a consequence, the product Cl_2_^+^ shows a propensity to be formed in the dissociative B^2^Σ_g_^+^ state with a bond order of 0.5. Since electrons populating both 3σ_u_* and 3σ_g_ molecular orbitals are mostly confined in the σ hole region, this peculiar feature of the chlorine molecule can be assimilated to a reaction catalyst. However, the formation of the fragment Cl^+^ requires a synchronization between the time required by an interacting complex to give an electronic rearrangement and the typical collision time. This synchronization is partially and totally relaxed with increasing *E*_coll_. Therefore, the Cl_2_^+^ production is predicted to increase with *E*_coll_, consistent with the experimental findings by Kischlat and Morgner^30^ and by our laboratory.^23^

It is intriguing to note that, under such conditions, the chemical (*direct*) mechanism is dominant and that it occurs through two basic steps: first, Cl_2_ undergoes a reduction to Cl_2_^–^ by CT in which the resulting neon behaves as an alkali atom (i.e., Na) as reducing agent (Redox 1 in Figure 3); in the second step, the Coulomb attraction promotes the trapping of the Ne^+^-Cl_2_^–^ ion pair at closer distances, where a concerted CT involving both internal 3σ_g_ and external 3σ_u_* populated molecular orbitals of Cl_2_^–^ (with the outer electron filling the p-orbital of the Ne and the other innermost electron being ejected) (Redox 2 in Figure 3), accompanied by molecular dissociation, determines the oxidation to the final state of Cl^+^. In this second case the Ne^+^ behaves like a halogen atom (i.e., F) as an oxidizing agent.

However, with increasing collision energy, the effectiveness of such a global mechanism, triggered by the Cl_2_ with the molecular axis aligned along R, decreases, since the collision time shortens, the passage through the crossing between neutral and ionic states assumes a less adiabatic character,^31^ and alignment effects are less probable. Under such conditions, collisions become statistically possible for all relative orientations of both partners, including the Cl_2_ molecule perpendicular approach to the Ne* atom and the global reactivity decreases. Here, in the perpendicular configuration of the formed adduct, rather unstable because of the absence of strong attractive components, both *indirect* (including possible radiative effects^9,32−34^) and *direct* (chemical or exchange) mechanisms become competitive, and above all an electron removal from the outer 3π_u_* molecular orbital becomes effective, leading to the single-step formation of Cl_2_^+^ in its ground electronic state X^2^Π_g_. This new channel increases the formation probability of Cl_2_^+^ with respect to Cl^+^.

### The NH_3_ Reaction Stereodynamics

**Figure 4:**
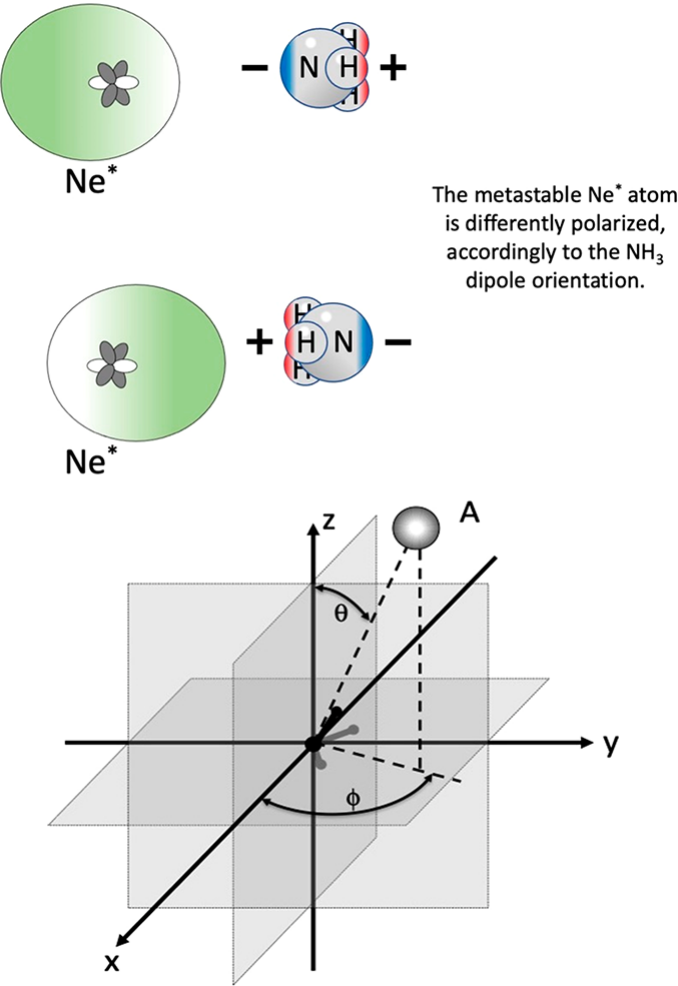
(lower panel) The polar coordinate system used to define the orientation of NH_3_ with respect to Ne*. (upper and intermediate panels) Two relevant configurations giving redox-reactive and nonreactive events. The metastable Ne* atom is differently polarized, accordingly to the NH_3_ dipole orientation. The positive charge density increase is approximately indicated by the increased extent of the red color; the corresponding change in negative charge density is likewise indicated in blue.

A system useful for the generalization of our approach is Ne*-NH_3_, for which a multidimensional potential energy surface (PES) given in analytical form^35^ assists in the formulation of the real part of the optical potential. A previous study,^25^ adopting a radial dependent imaginary part, whose average strength is modulated only by the NH_3_ orientation defined by the polar coordinates *R*, θ, ϕ given in the lower panel of Figure 4, suggested that CHEMI occurs exclusively on the N-side of the molecular frame. Specifically, while the Ne* approach within an angular cone confined around the *C*_3*v*_ ammonia axis promotes the formation of NH_3_^+^ in the X ground electronic state, the broadside approach in the vicinity of the perpendicular configuration leads to the formation of the electronically excited NH_3_^+^(A) ion that dissociates to NH_2_^+^ + H.^25^ The approach toward the hydrogen end of the molecule, along the *C*_3*v*_ ammonia axis, is accompanied by the strong polarization of the “floppy” cloud of 3s^1^ valence electron of Ne* that enhances the propensity to give an intermolecular hydrogen bond with a consequent reduction of the redox reaction effectiveness.^35^

The optical potential formulation, including also an effective angular-dependent imaginary Γ component, permitted us to estimate the acceptance of two angular cones where the reactions mainly occur. Details on the acceptance angular cones have been discussed in details in ref (25). However, no information has been provided on the relative role of *direct* and *indirect* mechanisms and therefore on partial ionization cross sections associated with the different reaction paths.

The present study, exploiting the analytical PES,^35^ attempts to deconvolve the effective imaginary part,^25^ separating the contributions from chemical and physical components of intermolecular forces, in order to identify the relative role of the two *direct* and *indirect* (basic) mechanisms. This preliminary objective is fundamental for characterizing the dependence of the relative role of the two basic reaction mechanisms on *E*_coll_ and therefore on the orbital angular momentum quantum number  of the collision complex that, in a classical picture, relates to the impact parameter *b*. Here, we analyze in detail two geometries of reagents approach, the one close to the *C*_3*v*_ molecular axis and the one in proximity of the perpendicular to this axis, that control the formation of NH_3_^+^ in the ground and first excited electronic states, respectively. Note that the two selected geometries are representative of the most probable configurations within the acceptance angular cones where the reaction occur, leading to the formation of a different type of ionic products. This allows the use of the same function for the imaginary Γ_1_ and Γ_2_ components, triggering *direct* and *indirect* mechanisms, respectively, whose general exponential formulation is borrowed from the atom–atom CHEMI reactions^6,7^ to describe *direct* and *indirect* mechanisms with their state-to-state dependence. In the present analysis, only the pre-exponential factors are adjusted to reproduce the magnitude and energy dependence of the total and partial ionization cross sections. Note also that any averaging over the angular acceptance cones is expected to change the pre-exponential factor values but not their ratios. The methodological choice of two selected configurations within the angular cones allows to highlight the different role of the centrifugal potential respect to the intermolecular interaction.

**Figure 5:**
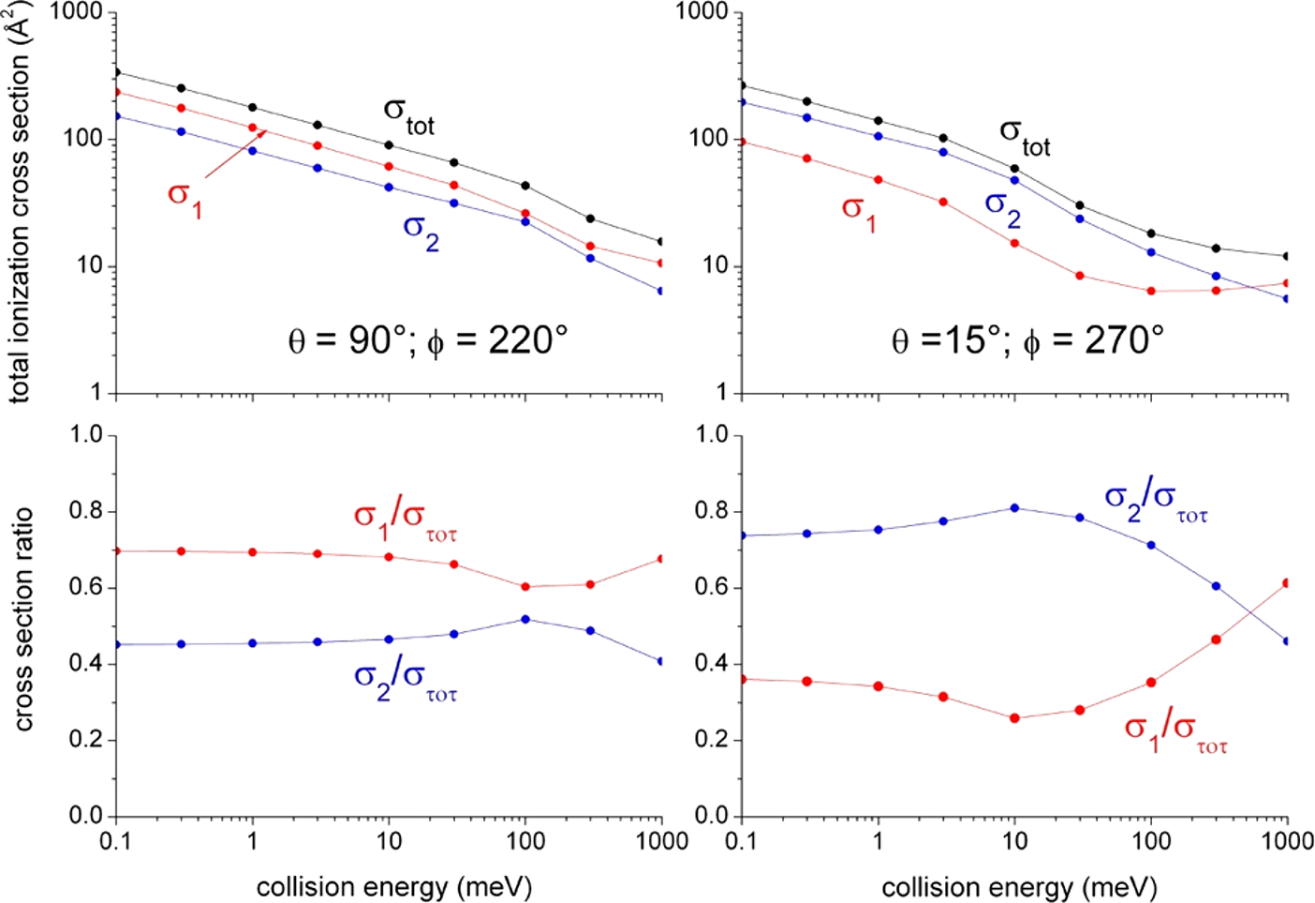
Collision energy dependence of partial (σ_1_, σ_2_) and total (σ_tot_) cross sections evaluated from individual (Γ_1_, Γ_2_) and total (Γ_tot_) components of the imaginary part (see Table 1) and referred to the two selected geometries. The subscripts 1 and 2 indicate the *direct* and *indirect* reaction mechanism contributions separately, respectively.

In previous work, the imaginary part of the optical potential was usually represented by a single effective radial component.^9,10,25^ Here, it is decomposed into two terms that control, as noted above, the selectivity and efficiency of the two basic *direct* and *indirect* mechanisms. Accordingly, the term Γ_1_ is related to the efficiency of the direct mechanism (exchange-redox), while Γ_2_ indicates the opacity function in the case of the *indirect* (radiative-photoionization) mechanism. Analogous symbolism is used for the cross sections calculated from the relative Γ_1_ and Γ_2_ functions as shown in Figure 5 (see below). Their separated formulation has been obtained according to the following guidelines:

(i) the quantities Γ_1_ and Γ_2_ must be related to intermolecular forces of a specific nature, whose strength scales in a different way with *R*. In particular, while chemical components, depending on the overlap integral between orbitals exchanging the electron, emerge at short separation distances and are strongly varying with *R*, those of physical origin show a much less radial dependence. Accordingly, completely different exponential functions have been adopted for the two imaginary terms, formulated as suggested from the detailed study of atom–atom reactions.^6,7^
(ii) Their relative and absolute strengths have been modeled in order to reproduce total and partial ionization cross sections in the right scale of experimental determinations. Considering the results provided by our^25^ and by another laboratory,^36^ cross sections represent a critical test of predicted values, since they cover 1–2 orders of magnitude and the probed *E*_coll_ varies for subthermal (∼0.1 meV) up to hyper-thermal values (−10^3^ meV), a changing of ∼4 orders of magnitude. More specifically, total ionization cross sections measured by the Losanna group^36^ vary from 300 to 400 Å^2^ (*E*_coll_ = 01 meV) up to ≃100 Å^2^ (*E*_coll_ = 10 meV), while those obtained in our laboratory cover a complementary range and are shown in Figure 1a.

Details of the selected geometries and on the formulation of the imaginary components are given in Table I.

**Table I:** Angular Coordinates Referred to the Two Selected Geometries and Formulation of the Individual and Total Imaginary Γ Components (in meV) as a Function of the Separation Distance *R* (in Å)^a^

	θ = 90°; ϕ = 220°	θ = 15°; ϕ = 270°
Γ_1_ (*R*)	3.0 × 10^5^ exp(−4.1 *R*)	3.0 × 10^5^ exp(−4.1 *R*)
Γ_2_ (*R*)	60.0 exp(−1.4*R*)	60.0 exp(−1.4*R*)
Γ_tot_(*R*)	Γ_1_ (*R*) + Γ_2_ (*R*)	Γ_1_ (*R*) + Γ_2_ (*R*)

a The Γ_1_ and Γ_2_ components for the two selected geometries of the approach of reagents within the acceptance angular cones (see text) have the same analytical formulation in order to highlight the different role of the centrifugal potential with respect to the intermolecular interaction potential.

**Figure 6:**
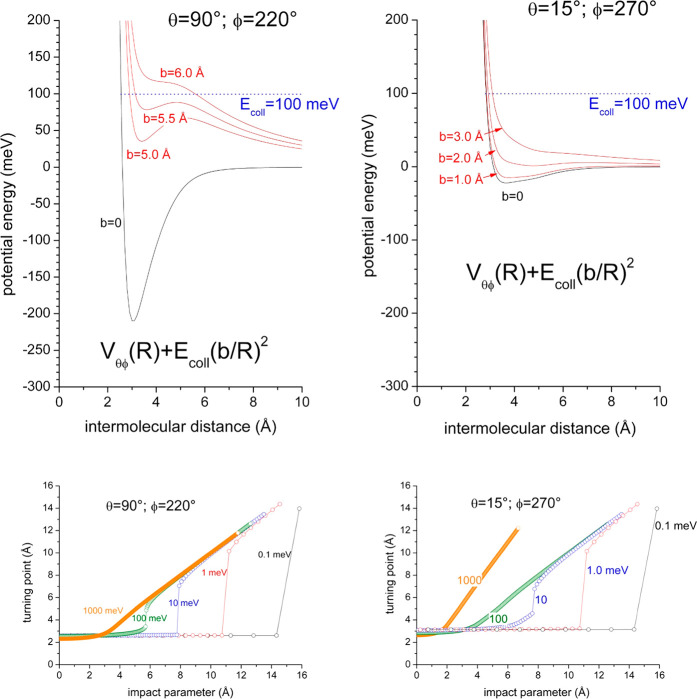
(upper panels) The dependence on the intermolecular distance *R* of the effective potential given as sum of the real component *V*_θφ_(*R*) and of the centrifugal contribution . (lower panels) The turning point dependence on the impact parameter *b*, or in the quantum picture on , evaluated for the two geometries at selected *E*_coll_ values that cover 4 orders of magnitude.

The total and partial (i.e., referred to each mechanism) ionization cross sections predicted by our method, and calculated within the semiclassical treatment, whose details can be found in refs (6) and (7), are shown in Figure 5. The results show that the role of two mechanisms and their energy dependence are completely different for the two geometries here considered, since the combination of the intermolecular potential and the centrifugal barrier selectively modulates the range of intermolecular distances probed so exalting the different role of *direct* and *indirect* mechanisms. In particular, for the geometry close to the *C*_3*v*_ NH_3_ axis (“collinear”), producing the NH_3_^+^(X) ground state, the *direct* mechanism is dominant at all *E*_coll_ values. However, for the “broadside” geometry, which gives rise to the formation of the NH_3_^+^(A) excited state with its subsequent dissociation into NH_2_^+^ + H, the *direct* mechanism becomes dominant only at hyper-thermal values of *E*_coll_. The contrasting behavior is ascribable to the different stability of the adduct formed in the two selected geometries for collision of reagents. Therefore, the detailed characterization of the dynamical evolution of the two types of collision complexes, leading to the turning points, which represent the most critical intermolecular distances where the reaction manifests the highest probability to occur, provides additional insight into critical features of the reaction stereodynamics. In particular, understanding the dependence of the turning points on *b* or on , that have been characterized, as emphasized in Figure 6, by a critical comparison between sum of real potential and centrifugal contribution with *E*_coll_, is of great interpretational value.

From the data reported in Figure 6 emerges the important selective role of the centrifugal barrier that generates, at collision energies lower than a critical *E*_coll_ value, turning points confined in well-separated ranges of values, where the reaction probability is completely different, while for higher collision energies a unique extended range of turning points becomes effective. As depicted in Figure 6, for the “collinear” geometry the critical *E*_coll_ value is ∼100 meV, while for the “broadside” geometry it amounts to ∼10 meV, and this variation arises from the different strength of the real potential that drives the collision. In particular, the centrifugal potential vanishes the trapping effect of the interaction more easily for the side approach because of the weaker attraction.

**Figure 7:**
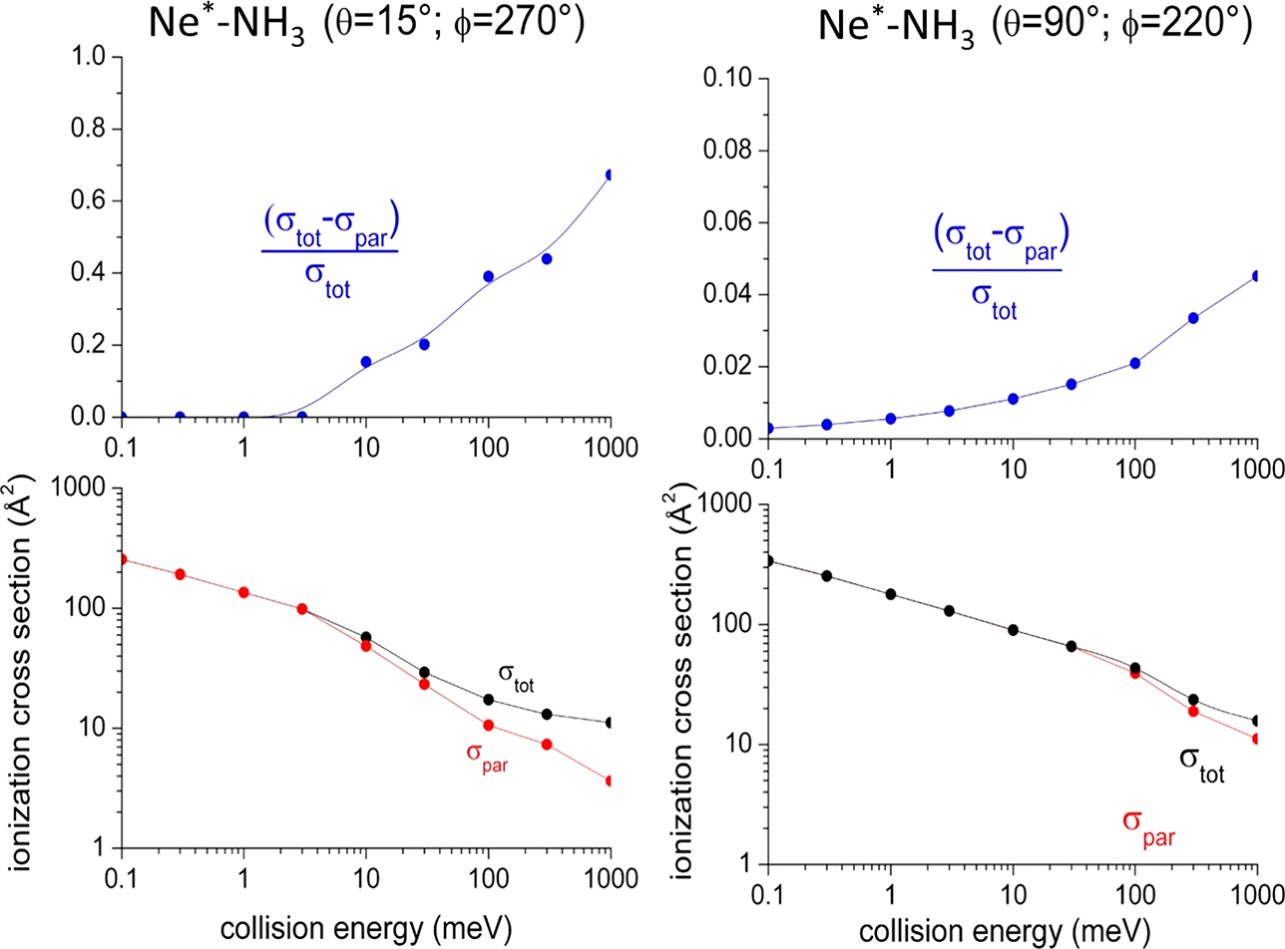
Total, σ_tot_, and partial, σ_par_, ionization cross sections determined by all  values and by , respectively.

The striking selectivity feature is that, for *E*_coll_ lower than the critical value, only a limited range of *b* or  values controls the reactivity. Moreover, under such conditions the collision time is sufficiently long, the phase accumulation along each reaction path depends on a similar passage from long to short *R* values, and then the relative role of the two mechanisms is approximately constant, as shown in Figure 5. Along these trajectories, the chemical reactivity can be also enhanced by the possible orientation of the polar molecule within the strong and anisotropic intermolecular field probe, favored by the low *E*_coll_ and by the long collision time. At higher *E*_coll_, in contrast, the range of turning points effective for reaction enlarges significantly, since the selective role of the centrifugal barrier (see upper panels in Figure 5), which separates short and large turning points, disappears. This is confirmed by the results in Figure 7, where, as a function of *E*_coll_, are plotted the total cross section (due to all *b* or  values contributions) and the partial cross section, determined exclusively by *b* or  lower than *b* or  values determining the absolute maximum of centrifugal barrier associated at each *E*_coll_ (*b*_max_ or , and for  values see Table II). In particular, at *E*_coll_ lower than the critical value, the centrifugal barrier completely separates the ranges of *b* or  driving the collisions (see Figure 6), making ineffective turning points determined by *b > b*_max_ or , since they occur at too large *R* values.

**Table II:** For the Two Selected Geometries, the Dependence of , Defining the Absolute Maximum of the Centrifugal Barrier, at Each Collision Energy

	θ = 90°; ϕ = 220°	θ = 15°; ϕ = 270°
*E*_coll_ (meV)	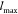	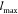
0.1	10	10
0.3	15	15
1	23	23
3	34	34
10	52	37
30	78	44
100	114	55
300	140	110
1000	190	115

Obtained cross-section values and their ratios demonstrate that the contribution from highest *b* or  values becomes appreciable only for *E*_coll_ larger than the critical value. Accordingly, the selective role of the centrifugal barrier tends to disappear, and a unique interval of *b* or  values promotes the reaction, making effective also those larger than *b*_max_ or , since determining turning points at intermediate and moderately large *R*.

## Conclusions

**Figure 8:**
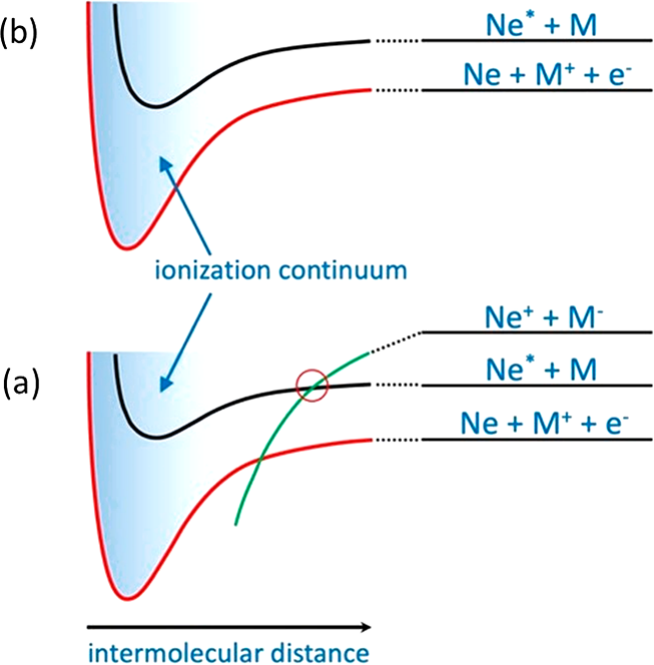
Scheme of the potential energy curves characterizing the two limiting cases for the *direct mechanism*. (a) CHEMI involving molecules with positive electron affinity (e.g., Cl_2_ and O_2_); (b) other cases mentioned in the text (e.g., NH_3_, N_2_, and CO).

It is important to stress that all stereodynamical effects emphasized for the CHEMI of Cl_2_ and NH_3_ must be considered averaged over all fine structure states accessible to the open-shell Ne*(^3^P_J_) reagent, identified for atom–atom reactions by *J* and Ω quantum numbers. Note that Ω provides the absolute value of the projection of the total (sum of the orbital and spin components) electronic angular momentum **J** along **R**, and it indirectly defines also the alignment degree of the half-filled p-orbital of Ne*(^3^P_J_) reagent respect to **R**. As demonstrated for atom–atom CHEMIs,^6,7^ both real and imaginary parts of the optical potential are depending on *J* and Ω, and this determines the opening of different state-to-state reaction channels. Figure 8 summarizes some basic differences between CHEMI reaction dynamics involving molecules in terms of a qualitative scheme of the potential energy curves that characterizes (see previous sections) two limiting cases for the *direct mechanism*. Figure 8a concerns molecules with positive electron affinity (e.g., Cl_2_ and O_2_), while Figure 8b concerns the other cases (e.g., NH_3_, N_2_, and CO).

For CHEMIs of molecules, the characterization of state-to-state reaction channels, with their dependence on both atomic alignment and molecular orientation, will be the target of a future extension of our methodology. Particular attention must be further addressed to the N_2_ and O_2_ reagents for which differences in the collinear and perpendicular approach of the diatomic molecule to Ne* are expected to emphasize a new selectivity in the reaction dynamics. In particular, while N_2_, from a phenomenological point of view (see Figure 1), behaves similarly to CO and CH_4_, with a total ionization cross section that increases with *E*_coll_, under thermal collision energies the cross section of O_2_ is at least a factor 3 larger with respect to that of N_2_, and it decreases with *E*_coll_ as for Cl_2_ and C_2_H_2_. The present paper suggests that the different behavior in the ionization cross sections of CHEMI involving molecules probably arises from a different balance of the intermolecular forces involved, which selectively depend on the fundamental physical/chemical properties of the molecules. In particular, while the electronic polarizability is comparable for N_2_ and O_2_, the electric quadrupole moment (that of N_2_, depicted in Figure 2, is approximately a factor 4 larger with respect to that of O_2_), energetics, and symmetry of highest occupied molecular orbital (HOMO) and lowest unoccupied molecular orbital (LUMO) molecular orbitals are completely different in the two cases.

Finally, it is interesting to note for Ne*-N_2_ important features of the isotropic optical potential were obtained from a multiproperty analysis of several experimental findings.^37^ In such a study the use of a combination of two imaginary components was necessary to reproduce simultaneously all analyzed experimental observables, which were probing complementary details of the interaction. According to the suggestions of this paper and our recent studies,^6,7^ this necessity probably relates to the occurrence of two competitive reaction mechanisms.

Therefore, the investigation from a phenomenological point of view of CHEMI reactions of prototype diatomic and polyatomic molecules emphasizes again the importance of experiments performed under single collision conditions and addressed to measure both ionization cross sections and PIES. The combined analysis of the experimental findings, that must be carried out adopting a proper formulation of the leading interaction components driving the collision dynamics, is then crucial to define the relative role of *direct* and *indirect* reaction mechanisms as a function of the geometry of the reagents approach and of the collision energy. The analysis of the reaction stereodynamics has allowed us to highlight important details on the microscopic redox mechanism of CHEMI, which is strongly dependent on the fundamental intrinsic characteristics of the target molecule and on the specific intermolecular interactions existing between the colliding partners, both crucial aspects in determining the formation of the transition state of the reaction. Our treatment is able to fully describe such reactions passing over the range from high temperature to ultracold collisions. This highlights the fact that that the “canonical” chemical oxidation process, dominant at a high collision energy, changes its nature in the subthermal regime to a pure direct photoionization process.^7^ It also points out differences between the *cold* chemistry of terrestrial and interstellar environments and the *hot* one of combustion and flames.^11−13,38,39^
